# Single-Nuclei Multiome (ATAC + Gene Expression) Sequencing of a Primary Canine Osteosarcoma Elucidates Intra-Tumoral Heterogeneity and Characterizes the Tumor Microenvironment

**DOI:** 10.3390/ijms242216365

**Published:** 2023-11-15

**Authors:** Rebecca L. Nance, Xu Wang, Maninder Sandey, Brad M. Matz, AriAnna Thomas, Bruce F. Smith

**Affiliations:** 1 Scott-Ritchey Research Center, Auburn University College of Veterinary Medicine, Auburn, AL 36849, USA; rln0005@auburn.edu (R.L.N.); xzw0070@auburn.edu (X.W.); 2 Department of Pathobiology, Auburn University College of Veterinary Medicine, Auburn, AL 36849, USA; mzs0011@auburn.edu; 3 Department of Clinical Sciences, Auburn University College of Veterinary Medicine, Auburn, AL 36849, USA; bmm0007@auburn.edu; 4 Department of Nursing, Tuskegee University, Tuskegee, AL 36088, USA; ariannat.2019@gmail.com

**Keywords:** osteosarcoma, single-nuclei, sequencing, multiome, tumor, heterogeneity, canine, dog, oncology, 10× genomics

## Abstract

Osteosarcoma (OSA) is a highly aggressive bone tumor primarily affecting pediatric or adolescent humans and large-breed dogs. Canine OSA shares striking similarities with its human counterpart, making it an invaluable translational model for uncovering the disease’s complexities and developing novel therapeutic strategies. Tumor heterogeneity, a hallmark of OSA, poses significant challenges to effective treatment due to the evolution of diverse cell populations that influence tumor growth, metastasis, and resistance to therapies. In this study, we apply single-nuclei multiome sequencing, encompassing ATAC (Assay for Transposase-Accessible Chromatin) and GEX (Gene Expression, or RNA) sequencing, to a treatment-naïve primary canine osteosarcoma. This comprehensive approach reveals the complexity of the tumor microenvironment by simultaneously capturing the transcriptomic and epigenomic profiles within the same nucleus. Furthermore, these results are analyzed in conjunction with bulk RNA sequencing and differential analysis of the same tumor and patient-matched normal bone. By delving into the intricacies of OSA at this unprecedented level of detail, we aim to unravel the underlying mechanisms driving intra-tumoral heterogeneity, opening new avenues for therapeutic interventions in both human and canine patients. This study pioneers an approach that is broadly applicable, while demonstrating significant heterogeneity in the context of a single individual’s tumor.

## 1. Introduction

Osteosarcoma (OSA) is a highly malignant bone tumor occurring most often in pediatric and adolescent humans as well as large-breed dogs. It is extremely heterogeneous and aggressive, with poor survival rates for both species. The median survival time for dogs undergoing amputation of the affected limb in combination with chemotherapy is approximately one year after diagnosis, with most dogs succumbing to metastases [1]. Comparative genetic and gene expression studies have demonstrated a high degree of similarity between human and canine osteosarcoma [2]. Due to its similarities, canine OSA represents a powerful translational model for understanding human disease as well as designing and testing clinical therapeutics [2,3]. 

Tumor heterogeneity makes treatment difficult due to the evolution of cell subsets that impact tumor growth, metastasis, and drug resistance [4]. Both intrinsic and extrinsic factors contribute to tumor heterogeneity, including the accumulation of genetic mutations, epigenetic factors affecting cellular activity and identity, and microenvironmental influences such as cell-cell interactions [4]. Single-cell sequencing is a powerful approach to evaluating tumor heterogeneity by identifying various cell types and states within a tumor. Identifying the relative proportion of cells with aberrant transcription patterns may provide critical information regarding the potential effectiveness of therapies [5]. 

However, obtaining viable cells after tissue dissociation is a prerequisite for single-cell sequencing and represents a major limitation of this technology, particularly for difficult tissues such as osteosarcoma. Since OSA is derived from bone, it often contains bone matrix, which is very rigid and difficult to homogenize. The dissociation procedure selects for cells that survive this process, potentially excluding rare cell types, and may also result in RNA degradation. Furthermore, enzymatic and/or mechanical tissue dissociation may alter the cell’s phenotype by inducing a transcriptional stress response, resulting in artifacts upon sequencing [6,7]. In our laboratory, efforts to isolate single-cell suspensions from primary canine OSA, including various mechanical and enzymatic protocols, have resulted in poor cell viability unsuitable for single-cell sequencing. Additionally, the removal of dead cells to increase viability may not accurately reflect the true biology of the tumor due to the selection of surviving cells. 

In contrast, single-nuclei sequencing circumvents the cell viability challenge by lysing the cells to obtain nuclei. However, nuclei quality is a critical factor to consider, and nuclear membranes should appear intact and with minimal blebbing under high-power microscopy after isolation. Importantly and in contradistinction to single-cell, single-nuclei sequencing can be performed on frozen archived samples [8]. 

Gene expression sequencing of single-nuclei differs from single-cell in the information it provides. Single-nuclei sequencing captures polyadenylated RNA transcripts that are actively being transcribed in the nucleus, whereas single-cell sequencing captures all polyadenylated RNA within the cell’s cytoplasm. Despite these differences, single-nuclei sequencing has been shown to provide equivalent gene detection signatures and accurate cell identification while minimizing bias compared to single-cell sequencing [6,7,9,10]. 

10× Genomics uses a microfluidic-based approach to partition single nuclei into gel beads containing barcoded primers and enzymes. This technology allows single nuclei to be captured and barcoded so that after sequencing, reads can be traced back to the corresponding cell/nucleus. Identifying individual cell phenotypes and genotypes can reveal the inherent intra-tumoral heterogeneity, including both malignant and non-malignant cell populations, at high resolution. Furthermore, this approach provides insight into the composition of the tumor microenvironment (TME), including interactions between tumor, stromal, and immune cells [5]. 

Based on the capabilities of single-nuclei signaling, we tested the hypothesis that this approach could be used to both identify heterogeneity between osteosarcoma cells within a single canine tumor and that additional, tumor-associated cell types could also be identified with this technique. To date, this is the first study to use single-nuclei multiome sequencing, including ATAC (Assay for Transposase-Accessible Chromatin) and GEX (Gene Expression) sequencing, of a treatment-naïve primary canine osteosarcoma, to simultaneously capture the transcriptomic and epigenomic profiles in the same nucleus and to demonstrate both the heterogeneity within the tumor and the associated cell populations. 

## 2. Results

To explore the cellular heterogeneity and microenvironment of canine OSA, single-nuclei multiome (ATAC + Gene Expression, GEX) sequencing was conducted on a primary canine OSA tumor lesion obtained from a 7-year-old male Doberman Pinscher presenting to the Wilford and Kate Bailey Small Animal Teaching Hospital at Auburn University College of Veterinary Medicine. Samples taken for sequencing were obtained prior to chemotherapy or the development of macro-metastatic lung disease. Histopathology confirmed the tumor to be osteoblastic osteosarcoma. 

### 2.1. Quality and Dimensionality of the Single-Nuclei and Sequencing Data

**Figure 1:**
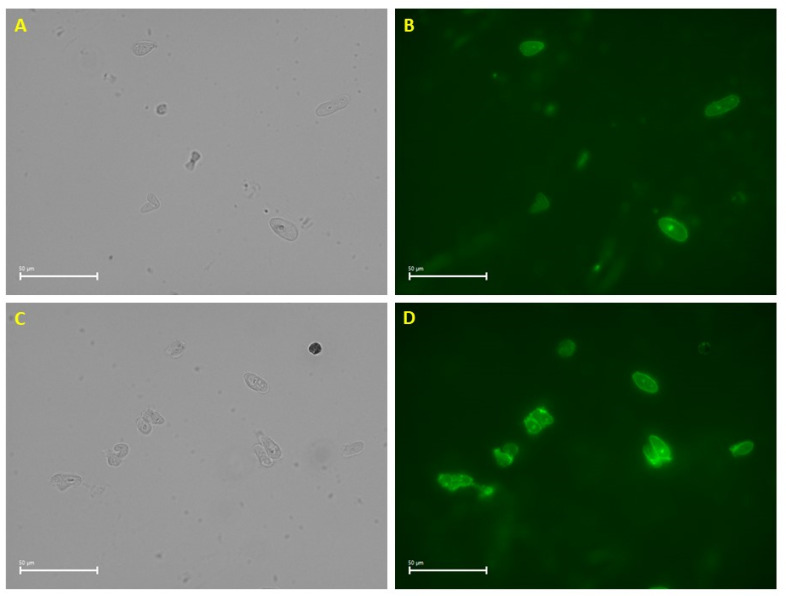
High-power microscopy to evaluate single-nuclei quality. Phase contrast (**A**,**C**) and AO/PI-stained nuclei under fluorescent microscopy (**B**,**D**) show that nuclear membranes are intact with minimal blebbing.

After isolation and prior to library preparation, nuclei were assessed for quality and quantity using high-power microscopy in combination with AO/PI fluorescent staining. The majority of nuclear membranes appeared intact with minimal blebbing (Figure 1). The nuclei were in sufficient quantity for library preparation, sequencing, and downstream analysis.

Approximately 10,000 nuclei were used for multiome (ATAC + GEX) library preparation using 10× Genomics technology and sequenced (150 bp PE) on the Illumina NovaSeq 6000 platform. After classification of each barcode into cell and non-cell groups, there were an estimated 5969 total nuclei sequenced with 8462 median ATAC high-quality fragments per cell and 2603 median GEX genes per cell. To examine true nuclei, filtering parameters (feature threshold 200–30,000 and count threshold 50–50,000) were applied to both ATAC and GEX data to eliminate empty droplets and doublets. After filtering, 5849 total nuclei and 23,784 covered gene models were included in the downstream analysis. 

### 2.2. Unsupervised Clustering to Evaluate Cellular Heterogeneity of Primary Canine OSA Reveals Nine Distinct Clusters

**Figure 2:**
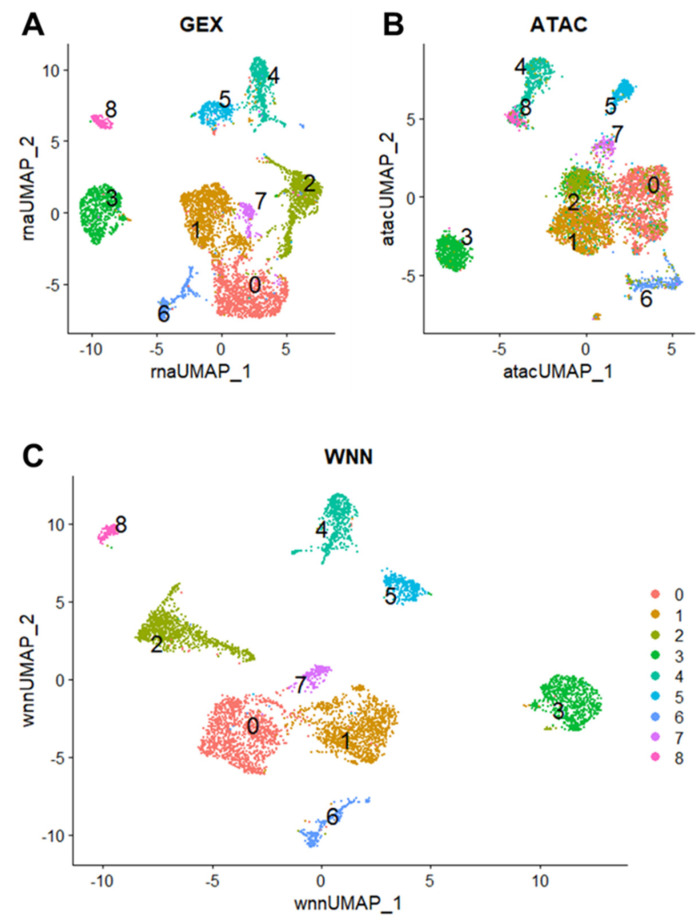
Cellular heterogeneity in primary canine OSA reflected by nine cell clusters. UMAP plot shows nine total clusters (c0–8) for GEX (**A**), ATAC (**B**), and weighted nearest neighbor (WNN) graph which combines both modalities (**C**). Each dot represents a single nucleus, and the color corresponds to the cluster.

Based on unsupervised clustering using principal component analysis (PCA) and graph-based dimensional reduction, we identified nine total cell clusters (c0–8) in the GEX (Figure 2A) and ATAC data (Figure 2B). The weighted nearest neighbor (WNN) procedure in Seurat v4 integrates multimodal data from the same cell to generate a unified representation of the dataset [11]. Using a weighted combination of the GEX and ATAC data, a WNN UMAP plot was generated to elucidate additional structure in the cellular clustering of canine osteosarcoma (Figure 2C). Clusters were numbered 0–8 and contained decreasing numbers of cells. For example, cluster 0 contained the most cells (1284/5849 cells, 21.9%), while cluster 8 contained the least number of cells (162/5849 cells, 2.8%) (Table 1). 

Clusters 0, 1, and 2 were more closely grouped and less discrete in the ATAC data, whereas the GEX data shows a more distinct relationship among these clusters (Figure 2A,B). These results suggest a relationship in the epigenetic programming of clusters 0, 1, and 2, despite differences in gene expression patterns. Conversely, the ATAC UMAP plot shows more separation between clusters 1 and 7, while the GEX UMAP plot shows a closer relationship and less clear distinction. This suggests that clusters 1 and 7 are closely related based on RNA expression but display different accessible motifs.

### 2.3. Cluster Annotation in Primary Canine OSA

**Figure 3:**
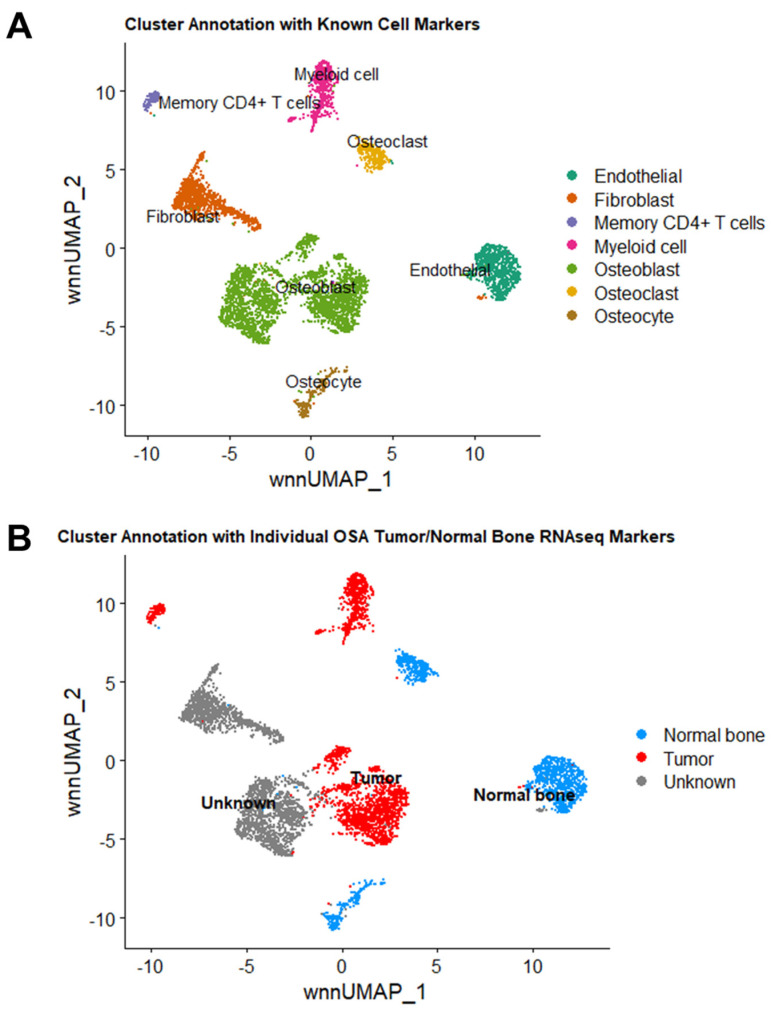
Cluster annotation with known cell markers and markers derived from differential analysis of bulk RNA sequencing of the same OSA tumor and patient-matched normal bone. Cell cluster annotation based on known single-cell marker genes (**A**) and marker genes from bulk RNA sequencing of the same primary OSA tumor and patient-matched normal bone (**B**). Each dot represents a single nucleus, and the color corresponds to the annotated cell group name.

To identify clusters based on cell type, a reference set containing single-cell markers for bone, osteosarcoma, and immune cells was used to annotate the clusters using ScType (Figure 3A) [12]. Osteoblasts were associated with clusters 0, 1, and 7 (2713/5849 cells, 46.4%); Fibroblasts were associated with cluster 2 (1023/5849 cells, 17.5%); Endothelial cells were associated with cluster 3 (798/5849 cells, 13.6%); myeloid cells were associated with cluster 4 (548/5849 cells, 9.4%); Osteoclasts were associated with cluster 5 (333/5849 cells, 5.7%); Osteocytes were associated with cluster 6 (272/5859 cells, 4.6%); and Memory CD4+ T cells were associated with cluster 8 (162/5849 cells, 2.8%) (Figure 3A, Table 1). 

To annotate the clusters based on “tumor” vs. “normal bone”, we generated an in-house annotation set using significantly up-regulated genes and down-regulated genes (adjusted *p*-value padj < 0.05 and fold-change FC <−2 and >2) derived from bulk RNA sequencing of the same primary canine OSA tumor and patient-matched normal bone generated in our previous study in Nance et al. (Figure 3B) [13]. Based on these results, tumor cells were related to clusters 1 (osteoblasts), 7 (osteoblasts), 4 (myeloid cells), and 8 (memory CD4+ T cells) (1591/5849 cells, 36.6%), which is consistent with the diagnosis of osteoblastic OSA. Normal bone was related to clusters 3 (endothelial cells), 5 (osteoclasts), and 6 (osteocytes) (1403/5849 cells, 24%). Clusters 0 (osteoblasts) and 2 (fibroblasts) had unknown relation to the bulk RNA seq data (2307/5849 cells, 39.4%) (Figure 3B, Table 1). 

Since bulk RNAseq is derived from a mixed population of cells, it is likely the sequenced tumor included tumor-initiating cells that drive tumor formation, tumor-associated cells such as fibroblasts and immune infiltrates, and a small proportion of normal bone cells such as osteoclasts and endothelial cells. Therefore, we cannot distinguish between tumor cells that drive tumorigenesis vs. those that are passengers in the process based solely on these results. It is apparent, however, that bulk RNA sequencing captures less than 40% of the tumor’s total cell population compared to single-nuclei RNA sequencing. 

Cluster annotation for each cell type was further inspected by plotting the expression of several marker genes derived from the CellMarker2.0 database that were used for annotation with ScType (Supplemental Figure S1). Although this is not an exhaustive list of markers used for annotation, osteoblast markers included *RUNX2*, *CDH11*, *PCNA*, *ACAN*, *MKI67*, *TOP2A*, and *COL1A1*; Fibroblast markers included *LUM*, *DCN*, *VIM*, *THY1*, *FAP*, *PRRX1*, and *COL1A1*; Endothelial markers included *CDH5*, *PECAM1*, *EGFL7*, *CD93*, *ENG*, and *EMCN*; Myeloid markers included *CD14* and *CD74*; Osteoclast markers included *ATP6V0D2*, *DCSTAMP*, *CTSK*, *OCSTAMP*, *MMP9*, and *ACP5*; Osteocyte markers included *GBLAP* (osteocalcin), *SPP1* (osteopontin), *CD86*, and *IBSP* (bone sialoprotein); Memory CD4+ T-cell markers included *CD3E*, *CD3D*, *CTLA4*, *LCK*, *LTB*, and *CD2* (Supplemental Figure S1). A full list of markers used for annotation is included in Supplemental Table S1. 

### 2.4. Copy Number Variation of Osteoblastic Clusters

**Figure 4:**
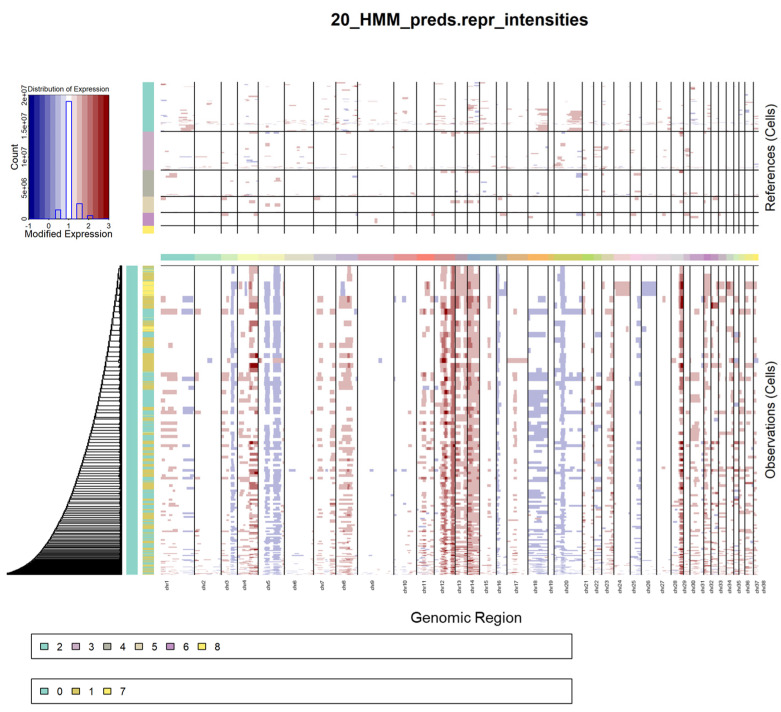
Heatmap of CNVs in osteoblastic clusters. Heatmap of the CNVs identified in the osteoblast clusters 0, 1, and 7 using the remaining clusters as the reference (A). Expression values for the normal cell clusters (depicted in the top heatmap) are subtracted from tumor cluster expression data (depicted in the bottom heatmap) to visualize differences. Rows are individual nuclei, columns are genes (ordered from left to right across the chromosomes); amplifications are colored red and deletions are colored blue.

OSA is characterized by significant genomic instability, resulting in large-scale chromosomal copy number variations. To evaluate the chromosomal structure, large-scale CNV analysis was inferred for the osteoblasts (clusters 0, 1, and 7) using the remaining clusters as the normal reference. Significant amplifications were observed in chromosomes 12–14 and deletions were present in chromosomes 5, 18, and 20 in the osteoblast clusters (Figure 4). Cluster 7 cells contain more CNVs compared to clusters 0 and 1 and their hierarchical relationship is reflected by the dendrogram. Compared to clusters 0 and 1, cluster 7 shows a distinct amplification of chromosome 24 and deletion of chromosome 26. 

### 2.5. Differentially Expressed Genes Define Clusters

**Figure 5:**
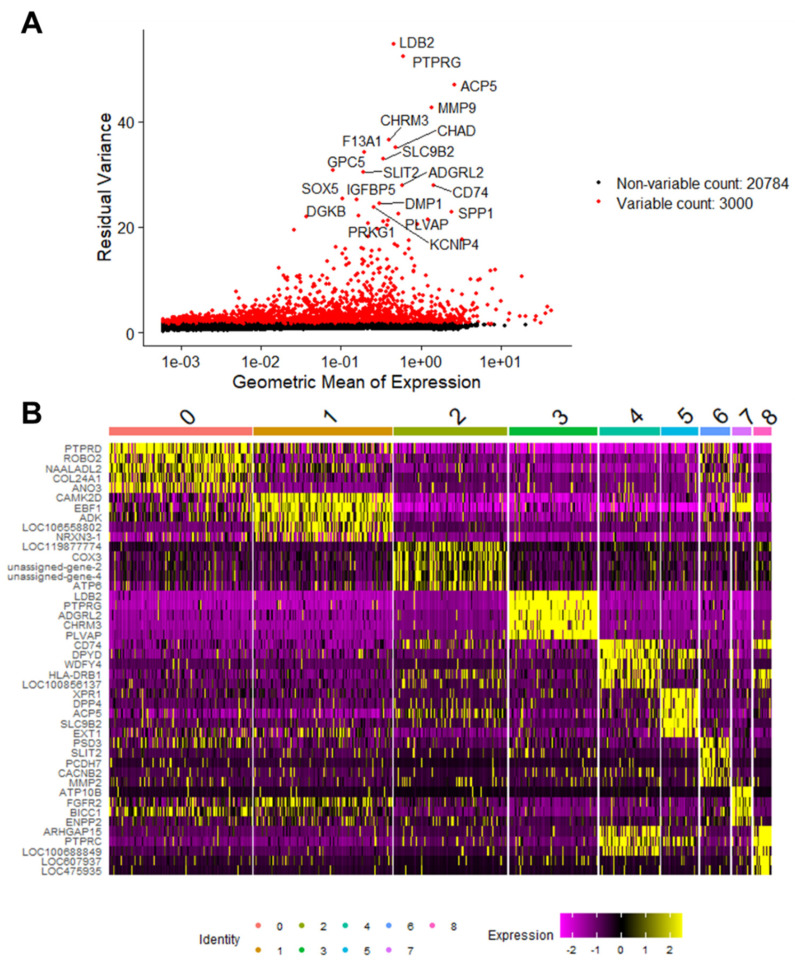
Differentially expressed genes define clusters in primary canine OSA. A plot of the top 3000 variable genes in the dataset with the top 20 most highly variable genes labeled. Red dots indicate the differentially expressed genes (*n* = 3000), and black dots represent the non-variable genes (*n* = 20,784) (**A**). Heatmap of the top 5 differentially expressed genes in each cluster. Clusters are identified by color and number on the top *x*-axis, gene symbols are listed on the *y*-axis; yellow indicates up-regulation and pink/purple indicates down-regulation (**B**).

After normalizing the UMI counts using a regularized negative binomial regression, highly variable features (genes) were identified to be used in downstream principal component analysis. Using the FindAllMarkers function in Seurat, markers were identified for every cluster compared to all remaining cells. The top 10 most highly variable genes according to the GEX data were *LDB2*, *PTPRG*, *ACP5*, *MMP9*, *CHRM3*, *CHAD*, *F13A1*, *SLC9B2*, *GPC5*, and *SLIT2* (Figure 5A). The top 5 differentially expressed genes defining each cluster were plotted on a heatmap (Figure 5B). Cluster 4 (myeloid), cluster 5 (osteoclasts), and cluster 8 (memory CD4+ T cells) share similar patterns of differential gene expression on the heatmap in Figure 5B, which is likely due to their related immunological functions and origins. 

### 2.6. Gene Set Enrichment Analysis Using Hallmark and Canonical Pathways

**Figure 6:**
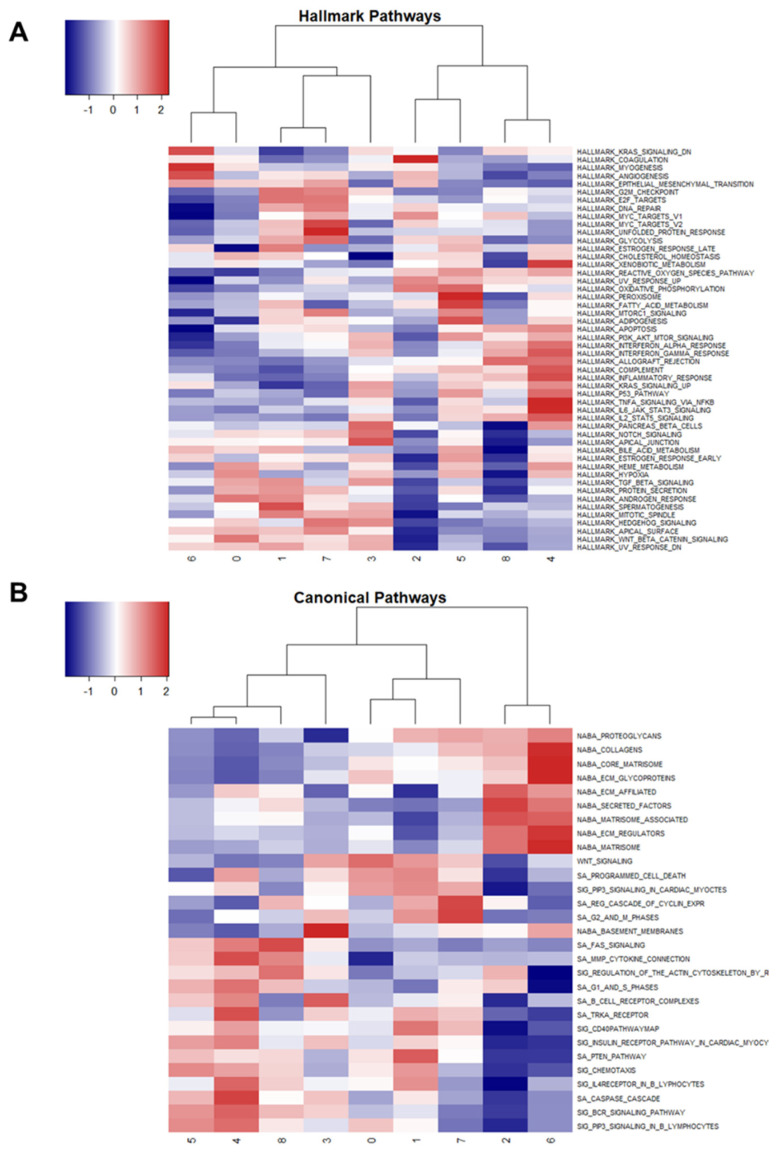
Gene set enrichment analysis among clusters using Hallmark and Canonical pathways. Heatmap of the Z-scores produced from gene set enrichment analysis using Hallmark Pathways (**A**) and Canonical Pathways (**B**). Red indicates up-regulation, and blue indicates down-regulation. Clusters are numbered on the *x*-axis.

Using the markers identified for each cluster, gene set enrichment analysis was performed using Hallmark and Canonical pathways to identify variation among clusters (Figure 6A,B). 

Interestingly, clusters 1 and 7 (tumorous osteoblasts) showed up-regulation of G2M transition, E2F targets, MYC targets v2, and glycolysis, but cluster 0 (osteoblasts with unknown relation to tumor/normal) showed down-regulation of these pathways. Cluster 0 also showed up-regulation of the hypoxia response. Collectively, these results suggest that clusters 1 and 7 consist of actively dividing osteoblasts driving tumor expansion, while cluster 0 may consist of necrotic and hypoxic osteoblasts. 

Cluster 8 (memory CD4+ T cells) and cluster 4 (myeloid cells) share similar patterns of Hallmark and Canonical pathway expression, likely due to shared immunological functions (Figure 6A). Similarly, cluster 5 (osteoclasts) and cluster 4 (myeloid cells) display similarities in Hallmark and Canonical pathways, which is explained by their shared macrophage functions. 

Cluster 2 (fibroblasts) shared patterns of enriched Canonical pathways with cluster 6 (normal osteocytes), with the exception of Regulation of the Actin Cytoskeleton by Rho GTPases, G1 and S Phases, and Regulation Cascade of Cyclin Expression, which were all down-regulated in osteocytes relative to fibroblasts (Figure 6B).

Collectively, the enriched Hallmark and Canonical pathway results support the clustering and annotation results by confirming shared relationships and functions among common cell types and aid in the elucidation of osteoblast heterogeneity. Cluster 0 osteoblasts show distinct down-regulation of cell cycle and up-regulation of hypoxia pathways in comparison to osteoblasts in clusters 1 and 7, which suggests that the largest cell cluster identified is perhaps responding to the body’s natural anti-tumor response. Furthermore, targeting this cluster of cells alone would likely not produce an effective response because these cells are not contributing to the active expansion of the tumor. 

### 2.7. Enriched Pathway Analysis Using GO Biological Processes

**Figure 7:**
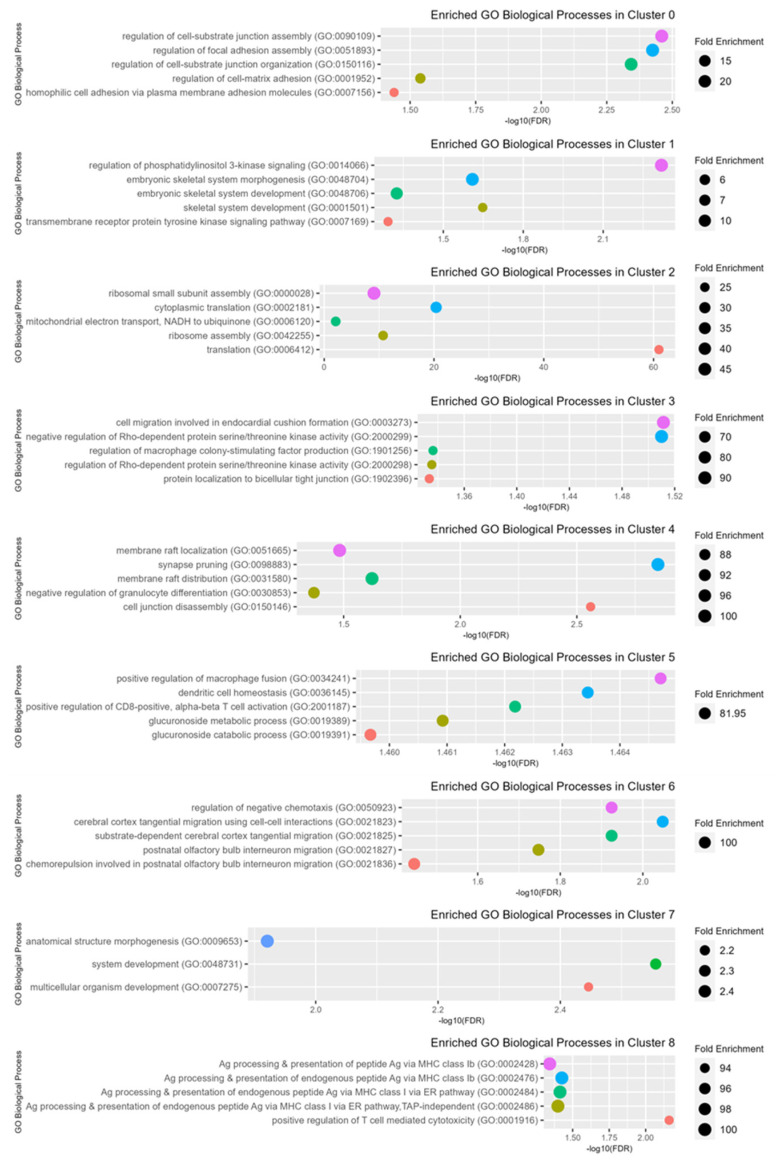
Enriched GO Biological Processes among clusters. The top 5 enriched GO Biological Processes and their associated fold enrichment and false discovery rate (FDR) for each cluster. Dot size correlates to the corresponding fold enrichment.

Using all genes in the canine genome database as a reference, a statistical overrepresentation test was performed on the significantly up-regulated genes from each cluster to identify enriched pathways based on GO Biological Processes. Using a false discovery rate (FDR) cut-off of 0.05, the top five pathways based on fold enrichment for each cluster are depicted in Figure 7. 

Enriched GO Biological Processes in cluster 0 osteoblasts included several pathways involved in the regulation of cell adhesion. Cluster 1 osteoblasts were enriched for regulation of PI3K signaling, skeletal system development, and transmembrane receptor protein tyrosine kinase signaling. Up-regulated pathways in cluster 2 (fibroblasts) were related to increased cellular activity and protein production, including ribosomal assembly, mitochondrial electron transport, and translation. Cluster 3 (normal endothelial cells) was enriched for negative regulation of Rho-dependent protein serine/threonine kinase activity, regulation of macrophage colony-stimulating factor production, and cell migration involved in endocardial cushion formation (a specialized region of mesenchymal cells that give rise to heart structures). The up-regulated GO Biological processes in cluster 4 (myeloid cells) included membrane raft localization/distribution, synapse pruning, negative regulation of granulocyte differentiation, and cell junction disassembly. Enriched pathways for osteoclasts in cluster 5 included macrophage fusion, dendritic cell homeostasis, positive regulation of CD8+ T cells, and glucuronoside metabolic/catabolic processes. Cluster 6 (normal osteocytes) up-regulated processes included regulation of negative chemotaxis and cell-cell interactions and migration. Cluster 7 (tumor osteoblasts) was enriched for pathways related to anatomical structure and system/organism development. Enriched pathways for cluster 8 (memory CD4+ T cells) were related to antigen processing and presentation via MHC class I and positive regulation of T-cell-mediated cytotoxicity (Figure 7).

### 2.8. Sub-Clustering and Elucidation of the Immune Cell Population 

**Figure 8:**
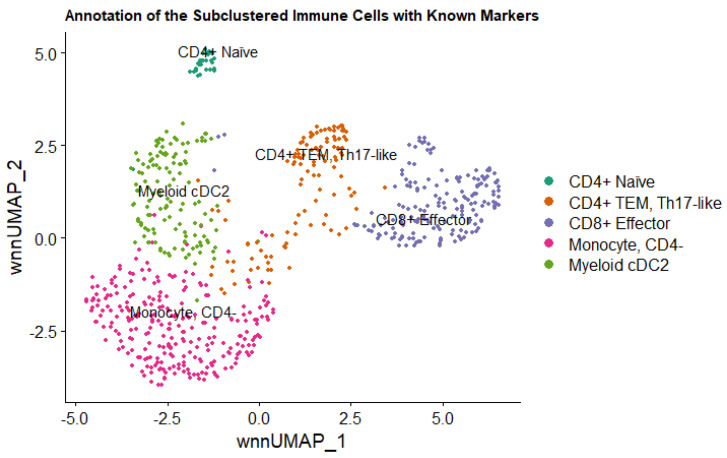
Annotation of the Immune Subclusters with Canine Leukocyte Markers. Sub-clustering and annotation of the immune cells present in the tumor microenvironment using known canine leukocyte markers reveals five distinct subclusters. Each dot represents a single nucleus, and the color corresponds to the annotated cell group name.

Clusters 4 (myeloid cells) and 8 (memory CD4+ T cells) were jointly subclustered to further characterize the immune population present in the tumor microenvironment (710 nuclei total). Principal component analysis using multimodal data (ATAC + GEX) revealed five subclusters of immune cells (Figure 8). The sub-clusters were annotated using a reference set derived from Ammons et al., 2023, which established a single-cell RNA sequencing atlas of circulating leukocytes in canine osteosarcoma and healthy controls [14]. The largest population was identified as CD4- Monocytes (262/710 cells, 36.9%). The second most abundant immune subcluster was identified as CD8+ Effector cells (171/710 cells, 24.1%), followed by myeloid cDC2 cells (131/710, 18.4%), CD4+ TEM, Th17-like cells (115/710, 16.2%), and CD4+ Naïve cells (31/710, 4.4%) (Figure 8). 

### 2.9. Comparison to Bulk Transcriptomic Sequencing Including the Same Tumor

In addition to characterizing the single-nuclei sequencing results of this tumor, we also sought to compare these results to bulk RNA sequencing of the same patient’s tumor. Our lab has previously published bulk RNA sequencing results of the same patient’s tumor along with six additional canine OSA tumors and patient-matched normal bone. The results, published by Nance et al., 2022, provide individual log2 fold-change values for each dog, in addition to bulk differential gene expression analysis. The patient belonging to the current study corresponds to patient C in the aforementioned manuscript [10].

**Figure 9:**
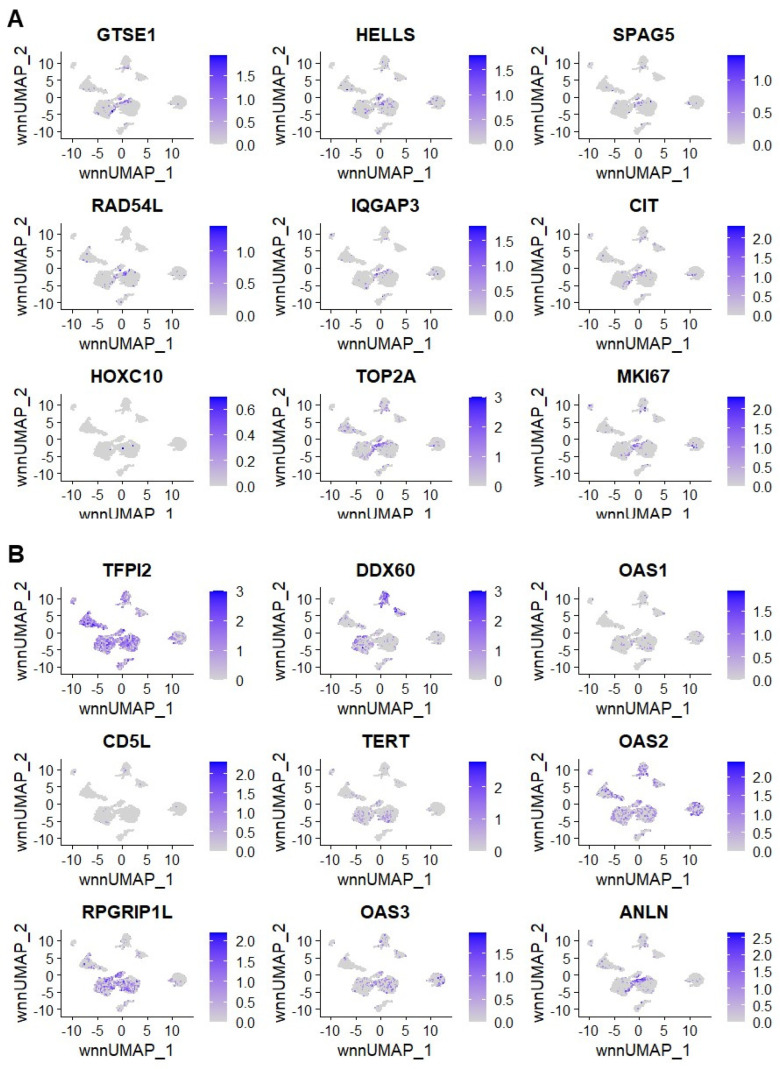
Expression of the top up-regulated genes from the bulk OSA tumor/normal bone RNAseq results. Expression of marker genes derived from the bulk OSA tumor/normal RNAseq dataset for the top up-regulated genes in the group (**A**). Marker gene expression for the top up-regulated genes in the same patient from the bulk OSA tumor/normal RNAseq dataset (**B**). Darker shades of purple indicate up-regulated expression, and gray indicates zero change.

The top up-regulated genes in OSA tumor compared to normal bone based on bulk RNA sequencing included *GTSE1*, *HELLS*, *SPAG5*, *RAD54L*, *IQGAP3*, *CIT*, *HOXC10*, *TOP2A*, and *MKI67* (Figure 9A). The top up-regulated genes in this patient based on bulk RNA sequencing of tumor and patient-matched normal bone included *TFPI2*, *DDX60*, *OAS1*, *CD5L*, *TERT*, *OAS2*, *RFGRIP1L*, *OAS3*, and *ANLN* (Figure 9B). Based on these results, the marker genes based on individual-level analysis capture more of the tumor’s heterogeneity than marker genes derived from bulk RNA sequencing. These results serve to validate our previously published approach to individual-level analysis using bulk RNA sequencing of tumor and patient-matched normal.

## 3. Discussion

To our knowledge, this is the first study to utilize single-nuclei multiome (ATAC + GEX) sequencing to characterize the molecular landscape of a treatment-naïve primary canine OSA tumor. Additionally, the results were compared to bulk RNA sequencing including the same tumor and patient-matched normal bone to further evaluate intra-tumoral heterogeneity.

Since osteosarcoma is a difficult tumor to homogenize, a modification was made to the 10× Genomics nuclei isolation protocol which resulted in high-quality nuclei suitable for sequencing. This protocol variation included the use of a bladed homogenizer in 0.5 strength Lysis buffer (included with the 10× Genomics kit). Upon observation with fluorescent dye, the nuclear membranes appeared mostly intact with minimal blebbing. Despite the recommendations from 10× Genomics to store the samples long-term in liquid nitrogen, storing them at −85 °C did not appear to affect the quality of our results. While approximately 10,000 nuclei were subjected to library preparation, only 5969 nuclei were sequenced. This is expected due to the microfluidic partitioning process which relies on dilution to prevent multiple nuclei in one droplet [15].

Unsupervised clustering and weighted nearest neighbor analysis identified nine cell clusters in primary canine OSA. As expected, the most abundant cell type present was osteoblasts, though these cells formed three distinct subclusters (clusters 0, 1, and 7). The second most abundant cell cluster contained fibroblasts (cluster 2) followed by endothelial cells (cluster 3). The tumor immune microenvironment included myeloid cells (cluster 4), osteoclasts (cluster 5), and memory CD4+ T cells (cluster 8). Similarly, many studies using single-cell RNA sequencing of primary and recurrent human OSA tissues identified nine major cell types, including osteoblasts, osteoclasts, fibroblasts, endothelial cells, myeloid cells, NK/T cells, B cells, and plasmocytes [16,17,18]. Our results are consistent with those found in human OSA, suggesting strong similarities in the intra-tumoral heterogeneity between canine and human OSA. However, caution should be exercised when making direct comparisons as treatment for human OSA typically involves intervention such as chemotherapy prior to sample acquisition and sequencing. In contrast, limb amputation usually occurs prior to any treatment in canine OSA, which may affect the cellular composition within the microenvironment due to clonal evolution.

Cluster annotation using up-regulated genes from bulk RNA sequencing of primary canine OSA and patient-matched normal bone resulted in the identification of cluster 1 (osteoblasts), cluster 7 (osteoblasts), and cluster 8 (memory CD4+ T cells) as OSA tumors while cluster 3 (endothelial) and cluster 6 (osteocytes) were associated with normal bone. Clusters 0 (osteoblasts), 2 (fibroblasts), 4 (myeloid), and 5 (osteoclasts) had unknown relation to the tumor/normal bone annotation. The inability of the annotation package to distinguish osteoclasts (cluster 5) and myeloid cells (cluster 4) as normal bone derivatives could be due to the relatively low proportion of these cells in comparison to osteocytes present in normal bone matrix. Markers for endothelial cells and myeloid cells are predicted to be expressed at low levels in this RNA dataset due to the bone processing technique to remove bone marrow, which contains the majority of myeloid cells and osteoclasts. It is also plausible that normal, non-transformed cells have altered gene expression patterns in response to the tumor microenvironment and signals from surrounding cells. The GEX profile of a normal cell in a normal environment would likely differ from that of a normal, non-neoplastic cell within the TME.

Second, while cluster 0 did not reflect the pattern of previously bulk-sequenced OSA tumors, it is unlikely that cluster 0, which contains the most cells, contains normal osteoblasts due to the small percentage of osteoblasts in normal bone. Osteoblasts also undergo age-related decline, and these bone samples were obtained from geriatric dogs [19]. Furthermore, CNV analysis showed this cluster contains many large-scale chromosomal rearrangements. Hallmark and Canonical pathway analysis suggest these cells are hypoxic, necrotic, and not actively dividing. Therefore, we predict this cluster consists of dying osteoblastic tumor cells in response to the body’s apoptotic immune signals and intrinsic anti-tumor response. Alternatively, or perhaps in conjunction, the tumor could simply be outgrowing its blood and nutrient supply. On the other hand, the activities of clusters 1 and 7 indicate these cells are highly active and thus more likely to be contributing to tumor growth and expansion.

Osteosarcoma has been predicted to be a poorly immunogenic tumor and therefore immunotherapies have been ineffective at managing OSA [20]. Based on up-regulated pathway analysis, immune cells in the TME (cluster 8) are actively participating in tumor antigen presentation via MHC class Ib and stimulating T-cell mediated cytotoxicity. The consequence of these actions is perhaps the dying osteoblasts in cluster 0. However, more studies are needed to understand how and why some cells escape the immune response.

Despite their prevalence in the tumor microenvironment, the inability to distinguish fibroblasts as tumors using the bulk data could be due to the low sample size (*n* = 7) of the bulk RNA sequencing data. Fibroblasts are also present in a smaller proportion in comparison to osteoblastic OSA cells. This reflects a limitation in differential gene expression analysis of bulk RNA sequencing data, where differences are obtained from averages across the entire population. The top marker gene for cluster 2 fibroblasts was *TMSB10*, which encodes a protein involved in cytoskeleton organization and cell migration. According to The Human Protein Atlas (https://www.proteinatlas.org/ENSG00000034510-TMSB10/single+cell+type, accessed on 12 August 2023), TMSB10 is typically expressed at low frequency in fibroblasts. Many of the other top marker genes for cluster 2 were involved in ribosomal assembly and function, including *RPS14*, *RPLP1*, *RPL36*, *RPS11*, and *RPS28*. These results suggest this cluster consists of highly active cells that are generating and secreting a large number of products that modulate the surrounding tumor microenvironment, consistent with the activities of cancer-associated fibroblasts (CAFs). As a major component of the tumor microenvironment, CAFs secrete a variety of factors that play a key role in tumorigenesis, and their activation is predicted to be controlled via epigenetic regulation [21]. Factors secreted by fibroblasts have been shown to modulate osteoblasts and their extracellular matrix remodeling functions [22]. This dynamic relationship is reflected in the clustering of the ATAC data, where the osteoblast and fibroblast clusters are less defined, closely interconnected, and show significant overlap. However, additional analyses are needed to elucidate this relationship.

Compared to single-cell sequencing, bulk RNA sequencing of the same sample captures only about 28% of the tumor’s heterogeneity, highlighting one of the major limitations of bulk sequencing. On the other hand, single-cell/nuclei sequencing is quite expensive and remains a limiting factor in applying this technology. Nonetheless, single-nuclei multiome sequencing provides an unparalleled view into the TME and intra-tumoral landscape. This approach is critical to improving targeted therapies and patient outcomes. Although this study consisted of a single primary tumor, the computational framework can be applied to additional tumors and the data can be conveniently reanalyzed as novel computational tools are developed.

In summary, we have successfully applied single-nuclei multiome sequencing to characterize the intra-tumoral heterogeneity and immune landscape of a treatment-naïve primary canine osteosarcoma.

## 4. Materials and Methods

### 4.1. Patient/Sample Description

Osteosarcoma tissue was obtained from a 7-year-old male Doberman Pinscher presenting to the Wilford and Kate Bailey Small Animal Teaching Hospital at Auburn University for limb amputation. Importantly, the samples were obtained prior to chemotherapy, radiation, or evidence of pulmonary macro-metastatic disease. A sample, adjacent to the sample used for sequencing, was subjected to histopathology and confirmed to be osteoblastic osteosarcoma. The tumor specimen was diced into approximately 50 mg pieces, immediately flash-frozen in liquid nitrogen, and stored at −80 °C. This sample was stored at −80 °C for approximately 5 years prior to nuclei isolation.

### 4.2. Nuclei Isolation

Nuclei were isolated from 42 mg flash-frozen OSA tissue by following the nuclei isolation kit protocol from 10× Genomics (Pleasanton, CA, USA, CG000505 Rev A) with minor adjustments to enhance homogenization while retaining nuclear morphology. The Lysis buffer provided with the kit was diluted with phosphate-buffered saline (PBS) to 0.5 strength and the sample was briefly homogenized (1–2 s) using a bladed homogenizer on ice, followed by a 5-min incubation on ice. The nuclei isolation protocol from 10× Genomics was then followed according to the manufacturer’s directions. Nuclei were visualized and counted using trypan blue (ThermoFisher Scientific, Waltham, MA, USA) and ViaStain acridine orange/propidium iodide (AO/PI) (PerkinElmer, Waltham, MA, USA) to determine quality and quantity using the Keyence BZ-X810 microscope with 100× oil-immersion objective. Nuclei were counted by hand using a hemocytometer in addition to using the Biorad TC20 automated cell counter (Biorad, Hercules, CA, USA) to determine the concentration for library preparation.

### 4.3. Library Preparation

Approximately 10,000 nuclei were used to generate ATAC and gene expression libraries. Libraries were prepared according to the Chromium Next GEM single-cell multiome ATAC + gene expression user guide (10× Genomics, Pleasanton, CA, USA, CG000338 Rev F). Briefly, the single-nuclei along with a master mix, 10× barcoded gel beads, and partitioning oil are loaded onto the Chromium Next GEM Chip J to generate single-nuclei gel bead-in-emulsions (GEMs). Pre-amplification PCR was performed and the GEX and ATAC libraries were split for further processing separately. The prepared libraries were shipped to Novogene (Sacramento, CA, USA) for 150 bp paired-end sequencing on the Illumina NovaSeq 6000 platform using the sequencing parameters recommended by 10× Genomics.

### 4.4. Bioinformatic Processing, Dimensional Reduction, and Weighted Nearest Neighbor Analysis

The canine reference genome “canFam6”, also known as Dog_10K_Boxer_Tasha (GCF_000002285.5), was used to align and count reads using Cell Ranger (v7.1.0). Analysis was accomplished using the Cell Ranger ARC Count (v2.0.2) pipeline and the output was loaded into Seurat (v4.3.0) for further processing. Seurat was used to filter the data using a feature threshold (200 < *n* < 30,000) and counts threshold (50 < *n* < 50,000). The SCTransform function in Seurat was used to normalize and transform the GEX data using a regularized negative binomial regression model, as described previously [23]. Dimensionality reduction was accomplished using PCA and UMAP embedding was used to visualize clusters using Seurat. ATAC data were processed using latent semantic indexing (LSI), which combines term frequency-inverse document frequency (TF-IDF) normalization followed by singular value decomposition (SVD) of the top identified features. A weighted combination of the GEX and ATAC data was used to construct a weighted nearest neighbor (WNN) graph and clusters were identified using the SLM algorithm in Seurat.

### 4.5. Cell Cluster Annotation

ScType was used to annotate cell clusters based on a given reference set of up-regulated/down-regulated markers and designated cell types [12]. A custom annotation set was created using single-cell markers accessed from CellMarker2.0 and annotated using ScType [24]. Annotation of “tumor” vs. “normal” clusters was based on differential expression analysis of the bulk RNA sequencing results produced by Nance et al. [13]. This dataset included bulk RNA sequencing of 7 primary canine osteosarcoma tumors, including the tumor in the current study, along with patient-matched normal bone. Using this data, a custom annotation set was created to designate “tumor cells” from “normal cells” based on up-regulated genes (log2 fold-change > 2 and padj < 0.05) in tumor and bone, respectively. ScType was then used to annotate the cell clusters and overlay the results on the weighted nearest neighbor UMAP plot. All markers used for cluster annotation are listed in Supplemental Table S1.

### 4.6. Copy Number Variation (CNV) Analysis

Large-scale chromosomal copy number was inferred based on gene expression for the osteoblast clusters using the Bioconductor package inferCNV with cluster 2 (fibroblasts), cluster 3 (endothelial cells), cluster 4 (myeloid cells), cluster 5 (osteoclasts), cluster 6 (osteocytes), and cluster 8 (memory CD4+ T cells) as the normal reference [25].

### 4.7. Differential Gene Expression for Identification of Cluster Markers

Using the variance-stabilized GEX data, the ‘FindAllMarkers’ function in Seurat was used to identify positive markers for clusters compared to all remaining cells using the roc/standard AUC classifier test (min.pct = 0.25 and logfc.threshold = 0.25). The positive markers for each cluster were subjected to subsequent pathway analysis using all genes in the canine database as the reference.

### 4.8. Enriched Pathway Analysis

Gene set enrichment analysis using Hallmark and Canonical pathways in the Canis lupus familiaris genome was accomplished using the R package singleseqgset (v0.1.2.9000). To identify enriched GO Biological Processes among clusters, PANTHER (v17.0) was used to perform a statistical overrepresentation test (Fisher’s exact test with FDR correction) using the GO Ontology database (DOI: 10.5281/zenodo.6799722 Released 1 July 2022) [26,27].

## Figures and Tables

**Table 1:** Cluster annotation using marker genes.

Cluster	# of Cells	% of Total # of Cells	Bone/OSA/Immune Markers (ScType Score)	Bulk RNAseq Tumor/Normal Markers (ScType Score)
0	1284	21.9%	Osteoblast (992)	Unknown (−471)
1	1249	21.3%	Osteoblast (1452)	Tumor cells (917)
2	1023	17.5%	Fibroblast (1113)	Unknown (160)
3	798	13.6%	Endothelial cell (6914)	Normal bone cells (931)
4	548	9.4%	Myeloid cell (3622)	Tumor cells (1017)
5	333	5.7%	Osteoclast (5236)	Normal bone cells (736)
6	272	4.6%	Osteocyte (700)	Normal bone cells (1403)
7	180	3.1%	Osteoblast (325)	Tumor cells (203)
8	162	2.8%	Memory CD4+ T-cell (1364)	Tumor cells (126)

## Data Availability

The data used in this study have been deposited into the NCBI GEO repository under accession number GSE244116. Scripts for data processing and figure generation can be found at https://github.com/rln0005/OSA_snMultiomeSeq, accessed on 2 October 2023.

## Supplementary Materials

The following supporting information can be downloaded at: https://www.mdpi.com/article/10.3390/ijms242216365/s1.
