# Supplementary material for: Single-Nuclei Multiome (ATAC + Gene Expression) Sequencing of a Primary Canine Osteosarcoma Elucidates Intra-Tumoral Heterogeneity and Characterizes the Tumor Microenvironment
Source: Int J Mol Sci. 2023 Nov 15;24(22):16365. doi: 10.3390/ijms242216365 (PMC10671194; doi:10.3390/ijms242216365)

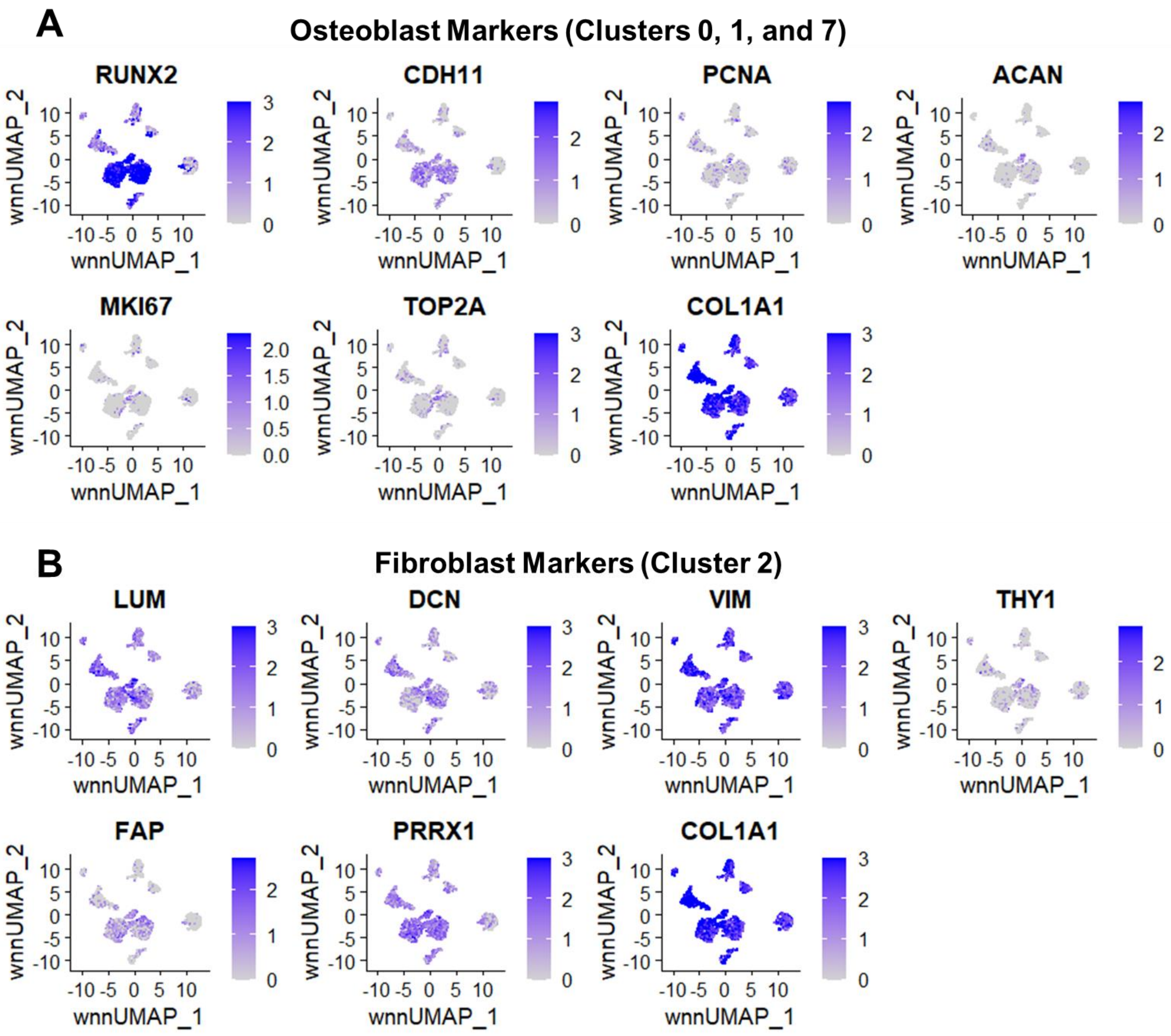
A
Osteoblast Markers (Clusters 0, 1, and 7)
RUNX2
CDH11
PCNA
ACAN
MKI67
TOP2A
COL1A1
B
Fibroblast Markers (Cluster 2)
LUM
DCN
VIM
THY1
FAP
PRRX1
COL1A1
wnnUMAP_1
wnnUMAP_2

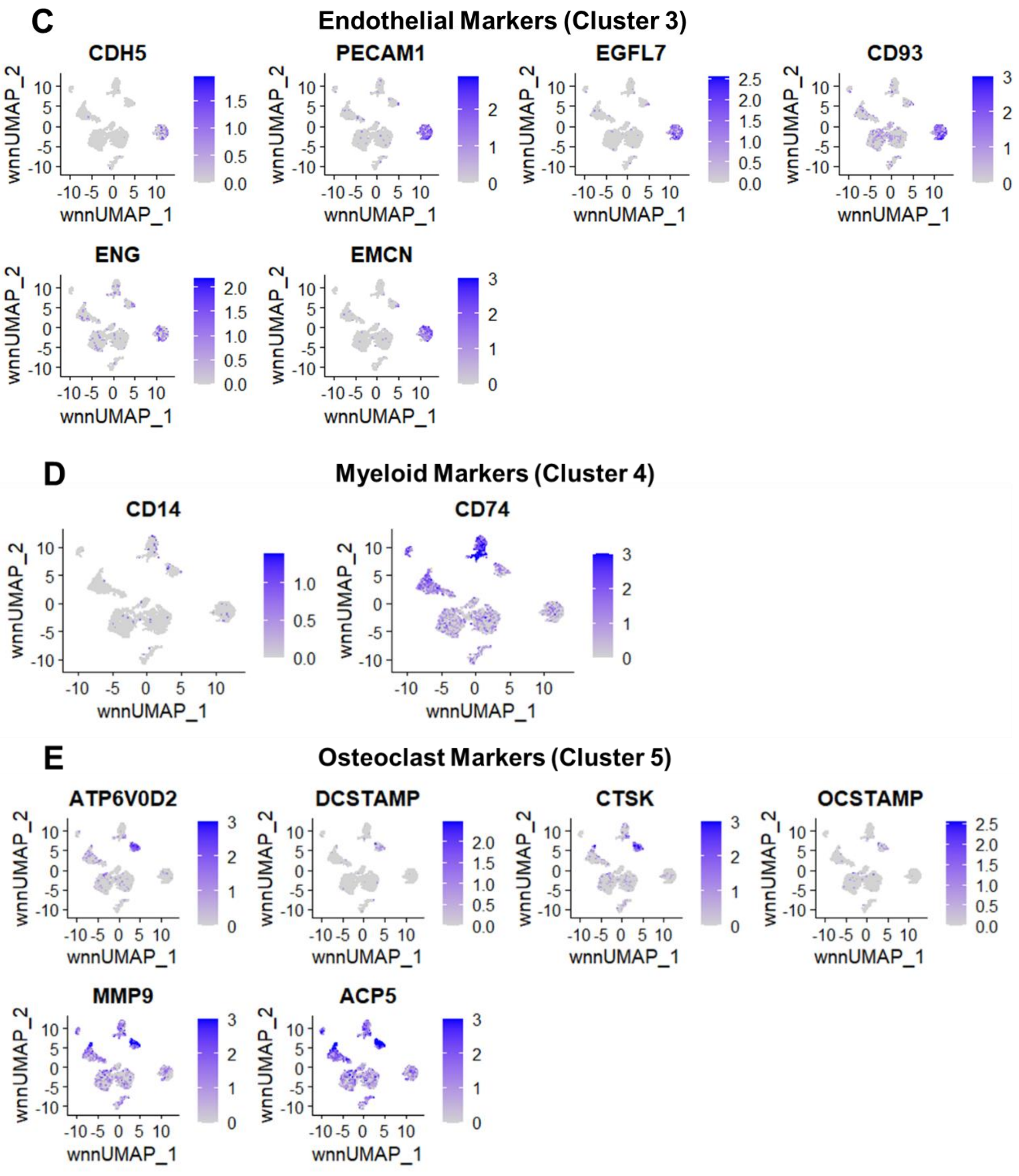

C
Endothelial Markers (Cluster 3)
CDH5
PECAM1
EGFL7
CD93
ENG
EMCN
D
Myeloid Markers (Cluster 4)
CD14
CD74
E
Osteoclast Markers (Cluster 5)
ATP6V0D2
DCSTAMP
CTSK
OCSTAMP
MMP9
ACP5
wnnUMAP_1
wnnUMAP_2

F **Osteocyte Markers (Cluster 6)**

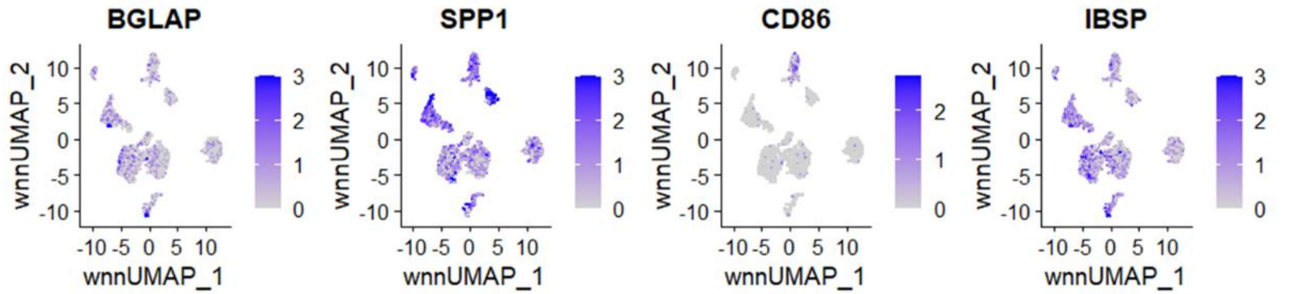

G **Memory CD4+ T cell Markers (Cluster 8)**

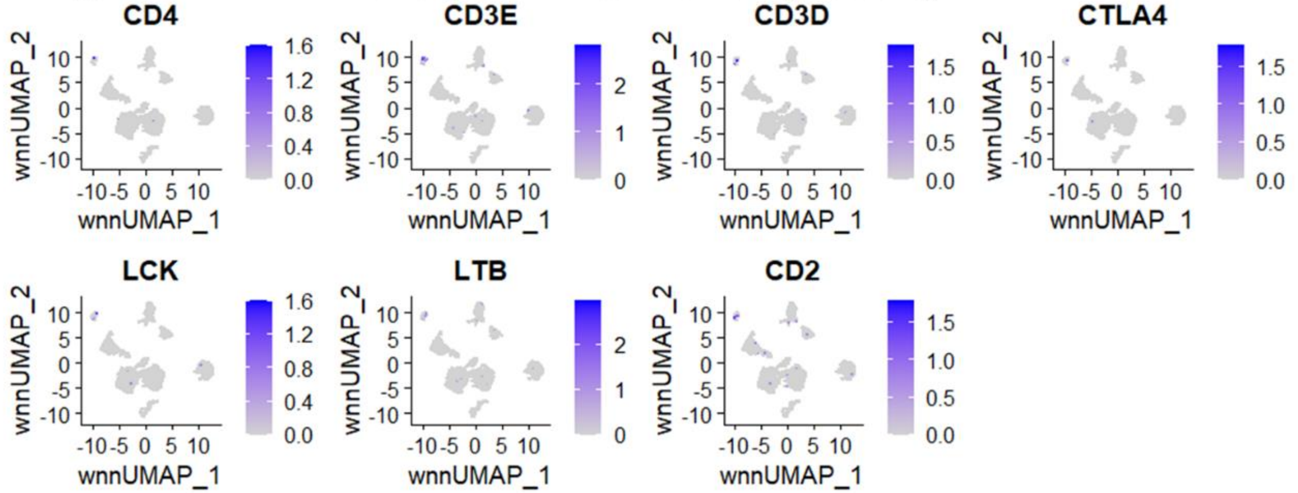

Supplement: Supplementary file 1 [file ijms-24-16365-s001.zip › Supplemental/SuppFig1.pdf]
